# The Severity of Sensorimotor Tracts Degeneration May Predict Motor Performance in Chronic Stroke Patients, While Brain Structural Network Dysfunction May Not

**DOI:** 10.3389/fneur.2022.813763

**Published:** 2022-03-31

**Authors:** Loukas G. Astrakas, Shasha Li, Sabrina Elbach, A. Aria Tzika

**Affiliations:** ^1^Department of Medical Physics, Faculty of Medicine, University of Ioannina, Ioannina, Greece; ^2^Department of Radiology, Athinoula A. Martinos Center of Biomedical Imaging, Massachusetts General Hospital, Harvard Medical School, Boston, MA, United States; ^3^NMR Surgical Laboratory, Department of Surgery, Massachusetts General Hospital, Harvard Medical School, Boston, MA, United States

**Keywords:** chronic stroke, Fugl-Meyer upper extremity scale, diffusion tensor imaging, sensorimotor cortex, graph analysis

## Abstract

Although the relationship between corticospinal tract (CST) fiber degeneration and motor outcome after stroke has been established, the relationship of sensorimotor cortical areas with CST fibers has not been clarified. Also limited research has been conducted on how abnormalities in brain structural networks are related to motor recovery. To address these gaps in knowledge, we conducted a diffusion tensor imaging (DTI) study with 12 chronic stroke patients (CSPs) and 12 age-matched healthy controls (HCs). We compared fractional anisotropy (FA) and mean diffusivity (MD) in 60 CST segments using the probabilistic sensorimotor area tract template (SMATT). Least Absolute Shrinkage and Selection Operator (LASSO) regressions were used to select independent predictors of Fugl-Meyer upper extremity (FM-UE) scores among FA and MD values of SMATT regions. The Graph Theoretical Network Analysis Toolbox was used to assess the structural network of each subject's brain. Global and nodal metrics were calculated, compared between the groups, and correlated with FM-UE scores. Mann–Whitney *U-*tests revealed reduced FA values in CSPs, compared to HCs, in many ipsilesional SMATT regions and in two contralesional regions. Mean FA value of the left (L.) primary motor cortex (M1)/supplementary motor area (SMA) region was predictive of FM-UE score (*P* = 0.004). Mean MD values for the L. M1/ventral premotor cortex (PMv) region (*P* = 0.001) and L. PMv/SMA region (*P* = 0.001) were found to be significant predictors of FM-UE scores. Network efficiency was the only global metric found to be reduced in CSPs (*P* = 0.006 vs. HCs). Nodal efficiency of the L. hippocampus, L. parahippocampal gyrus, L. fusiform gyrus (*P* = 0.001), and nodal local efficiency of the L. supramarginal gyrus (*P* < 0.001) were reduced in CSPs relative to HCs. No graph metric was associated with FM-UE scores. In conclusion, the integrity of CSTs connected to M1, SMA, and PMv were shown to be independent predictors of motor performance in CSPs, while stroke-induced topological changes in the brain's structural connectome may not be. A sensorimotor cortex-specific tract template can refine CST degeneration data and the relationship of CST degeneration with motor performance.

## Introduction

The quality of life of chronic stroke patients (CSPs), including their ability to live independently, to have active social lives, and to return to their professional occupation and pre-stroke daily activities, is highly dependent on severity of motor impairment and mobility (1). Recovery after stroke is a complex multiparametric process involving socio-demographic, clinical, and genetic factors (2). The location of the initial stroke-induced injury is a major clinical factor affecting motor function deficits and recovery potential (3). Indeed, the integrity of motor-related cortical areas and their descending tracts correlate with persistent motor impairment in the chronic stroke phase (4–7).

Diffusion tensor imaging (DTI) is a magnetic resonance imaging (MRI) technique that is widely used to assess changes in tissue microarchitecture and to probe structural integrity of the brain noninvasively (5, 8). Accordingly, DTI visualization of white matter (WM) tracts is useful for examining stroke-induced WM connectivity alterations (9). It has been established in both human and animal DTI studies that corticospinal tract (CST) damage and corticobulbar motor tract damage can produce profound motor deficits and determine the recovery course after stroke (6, 7, 10–14).

Most stroke studies applying DTI tractography have focused on the CST because of its central importance in motor function (10, 13, 15, 16). Commonly, CST reconstruction is conducted by pairing a subcortical seed region of interest (ROI) (e.g., anterior portion of upper pons, medulla) and a cortical ROI, usually the primary motor cortex (M1). Alternatively, a CST template can be used to define ROIs to be analyzed (15). These approaches are limited by the fact that they probe only a portion of descending tracts. Only about half of CST fibers originate in M1 (17) and motor function recruits many cortical areas, such as the premotor cortex and supplementary motor area (SMA), each with its own descending tracts to subcortical areas or to the spinal cord directly (18, 19). Although standard WM tract atlases (e.g., Johns-Hopkins WM template and the Harvard-Oxford cortical and subcortical structural atlas) show multiple tracts and their relationships with WM areas important to motor function, such as the posterior limb of the internal capsule (PLIC) (20), they do not provide detailed information about the cortical targets of WM tracts where motor information processing takes place. The only template that relates sensorimotor WM tracts to their cortical topography is the probabilistic sensorimotor area tract template (SMATT) (21). The probabilistic SMATT links stroke lesions with sensorimotor cortical processing directly and precisely, but has been used only rarely in stroke studies (22).

Although the initial stroke lesion is a major determinant of final motor outcomes, secondary degeneration and plastic structural brain changes, either spontaneous or induced by therapy, remodel the brain (23). Whole-brain adaptation following stroke involves remote regions in addition to perilesional areas (24). Structural MRI studies focused on specific brain regions do not provide information about global reorganization of the brain's connectome. Graph analysis, which includes measures of regional and global brain network characteristics, can provide information about whole-brain connectivity (25). According to graph theory, the brain can be represented graphically as a complex network of anatomical areas (nodes) and links (edges) that represent connectivity between the nodes. Structural graphs of the brain can be developed from DTI tractography data, usually by defining nodes as atlas regions and defining edges as the number of tracts connecting them. Graph analysis studies have revealed brain network aberrations in disorders such as Alzheimer's disease, schizophrenia, and Parkinson's disease (26). However, studies applying network approaches to assess brain connectome remodeling after stroke are still scarce (24, 27–29).

The aim of the present study was to use DTI with SMATT-based segmentation of the sensorimotor tracts in CSP and healthy control (HC) groups to assess how clinical motor outcomes are related to stroke-induced damage of WM tracts linked to sensorimotor cortical areas. We used graph analysis to find brain structural network abnormalities in CSPs and to assess whether these abnormalities are predictors of motor performance during recovery.

## Materials and Methods

### Subjects

Twelve CSPs (mean age, 52.7 ± 13.8 years; 4 men, 8 women) and 12 age-matched, right-handed HC volunteers (57.4 ± 11.3 years; 4 men, 8 women) participated in this study. Patients were recruited through the registries of stroke survivors at Massachusetts General Hospital. The inclusion criteria for CSPs were: (a) first-ever ischemic stroke incurred in the left middle cerebral artery territory at least 6 months prior to recruitment; (b) acute unilateral loss of hand strength score of <4 on the Medical Research Council scale for ≥48 h; and (c) right-handedness according to the Edinburgh Handedness Inventory. The exclusion criteria were: (a) the presence of any hearing, vision, language, or cognitive deficit; (b) MRI contraindications; and (c) any disorder that impairs motor function of the stroke-affected hand. Institutional review board approval of the study was granted by the Partners Human Research Committee (protocol no. 2005P000570). All participants provided informed consent. Patients' motor performance was assessed before imaging with the Fugl-Meyer Upper Extremity (FM-UE) scale for sensorimotor impairment. The Modified Ashworth scale was used to assess spasticity.

### Imaging

Brain scans were performed with a 3-T Skyra Siemens full-body scanner equipped with a 32-channel phased-array surface coil. The MRI protocol consisted of three sequences. The first was a sagittal magnetization-prepared rapid gradient-echo sequence for high-resolution, T1-weighted anatomical imaging with the following parameters: repetition time (TR)/echo time (TE)/inversion time (TI) = 2,300 ms/2.53 ms/900 ms; field of view (FOV), 256 mm; resolution, 1 × 1 × 1 mm^3^; PAT factor 2; and acquisition time, 5.5 min. The second was an axial fat-saturated single-shot spin-echo planar imaging sequence for diffusion imaging with b-values of 0 and 1,000 s/mm^2^ and the following parameters: voxel size 2 × 2 × 2 mm^3^; 69 slices; PAT factor 2; TR/TE 12,200 ms/104 ms; 30 diffusion directions; acquisition time, 6.9 min. Finally, we conducted three-dimensional (3D) fluid-attenuated inversion recovery pulse sequence for clinical evaluation with the following parameters: voxel size, 0.5 × 0.5 × 0.9 mm^3^; 192 slices; PAT factor 2; TR/TE/TI = 5,000 ms/386 ms/1,800 ms; and acquisition time, 5.6 min.

### Data Analysis

The variable stroke size refers to the stroke lesion size calculated semiautomatically with the itk-snap tool. The variable time after stroke refers to the time from the stroke incident and to the imaging scan. Associations of stroke size and time after stroke with FM-UE score and SMATT-region diffusion metrics were assessed with determining Spearman correlation coefficient rho values, applying Bonferroni correction for multiple comparisons.

Mean values are reported with standard deviations (SDs) or standard errors (SEMs) as indicated. Imaging data were analyzed in PANDA (Pipeline for Analyzing braiN Diffusion imAges), which is based on the FMRIB Software Library and Diffusion Toolkit (http://trackvis.org/dtk/). For each subject, we used the FMRIB Diffusion Toolbox to correct diffusion-weighted images for eddy current-induced distortion and motion artifacts. A brain mask was created from the first b0 image with the Brain Extraction Tool. The FMRIB Diffusion Toolbox was used to fit the tensor model and compute fractional anisotropy (FA) and mean diffusivity (MD) maps. The diffusion maps were normalized to the ICBM152 template as described by Gong et al. (30). For each subject, an FA image in native space was co-registered to its corresponding high-resolution T1-weighted structural image by way of affine transformation. Then the structural image was non-linearly registered to the template T1-weighted image. A warping transformation from the native to the standard space was obtained by combining the transformations in these two steps. Two standard-space atlases were then warped inversely back to individual native space *via* inverse transformation. One atlas, the probabilistic sensorimotor area tract template (SMATT), was used for ROI analysis. A second atlas, namely the Automated Anatomical Labeling atlas (AAL), was used for graph analysis of the brain connectome.

**Figure 1:**
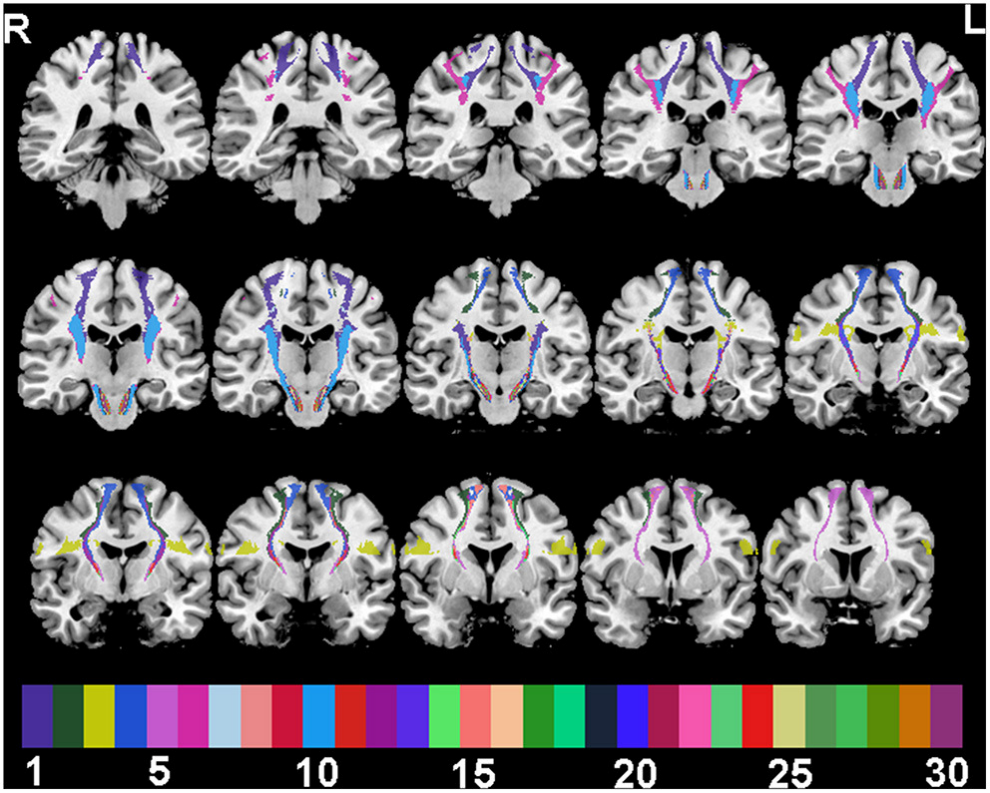
SMATT tracts in a random colour-scale superimposed on 15 grayscale coronal T1-weighted anatomical slices. Colour indices are: 1 M1; 2 PMd; 3 PMv; 4 SMA; 5 preSMA; 6 S1; 7 M1,PMd; 8 M1,PMv; 9 M1,SMA; 10 M1,S1; 11 PMd,PMv; 12 PMd,SMA; 13 PMv,SMA; 14 PMd,preSMA; 15 SMA,preSMA; 16 M1,PMd,SMA; 17 M1,PMv,SMA; 18 M1,PMv,S1; 19 M1,SMA,S1; 20 PMd,PMv,SMA; 21 M1,PMd,SMA,S1; 22 M1,PMv,SMA,S1; 23 M1,PMd,PMv,SMA; 24 PMd,SMA,preSMA; 25 M1,PMd,PMv,SMA,S1; 26 M1,PMd,SMA,preSMA; 27 PMd,PMv,SMA,preSMA; 28 M1,PMd,SMA,preSMA,S1; 29 M1,PMd,PMv,SMA,preSMA; 30 M1,PMd,PMv,SMA,preSMA,S1.

For ROI analysis, SMATT segments of corticofugal tracts were defined based on six motor-related cortical regions: M1, dorsal premotor cortex (PMd), ventral premotor cortex (PMv), SMA, preSMA, and primary somatosensory cortex (S1). Probabilistic SMATT further divided the 12 sensorimotor tracts (6 per hemisphere) into 60 tracts (30 per hemisphere) based on SMATT tract overlap (Figure 1). For example, the left (L.)-PMd,PMv,SMA region contains voxels of the L. hemisphere that have equal probability, 1/3, of belonging to the PMd, PMv, or SMA.

Mean values were calculated for probabilistic SMATT template regions in the native space for each diffusion metric maps. Shapiro-Wilk tests were used to determine normality and Mann–Whitney *U*-tests were used to assess group differences in diffusion metrics (HC vs. CSP). *P*-value threshold for the multiple comparisons was calculated with Bonferroni correction. Least Absolute Shrinkage and Selection Operator (LASSO) regressions were used to select independent FM-UE score predictors from among the diffusion metric mean values of numerous probabilistic SMATT regions. LASSO is a regularization technique that provides more accurate prediction than standard linear regression methods when there are more variables than data points. It improves a linear model's accuracy by minimizing the residual sum of squares similar to the minimization of standard linear regression. Additionally, it penalizes model complexity by minimizing the sum of absolute values of the regression coefficients. LASSO functions as a feature selection method by shrinking coefficients toward zero, with some becoming exactly zero. The balance between accuracy and complexity is determined by the λ coefficient. A 10-fold cross-validation approach was used to estimate λ and the optimal LASSO model. Bootstrapping with 1,000 times resampling was used to estimate standard errors of the predictor's coefficients.

In preparation for graph analysis, native FA maps were fed into the PANDA toolbox, which, using the Diffusion Toolkit (http://trackvis.org/dtk/), reconstructed all possible fibers within the brain. For each subject, a graph was then constructed with the AAL ROIs as nodes. Graph edges are represented with a number-weighted matrix M, where the element M(i, j) represents the number of fibers linking nodes i and j. In accordance with recent recommendations (31), M was not thresholded. Nodal metrics (clustering coefficient, shortest path length, efficiency, local efficiency, degree centrality, and betweenness centrality) and global network metrics (small-world, efficiency, rich-club, and modularity) were calculated with the Graph Theoretical Network Analysis Toolbox, version 20. These metrics were calculated and interpreted as described in detail elsewhere (32). Network metrics were compared between CSP and HC groups with *T*-tests, with age and gender as covariates. Associations between graph metrics and FM-UE scores were evaluated with Pearson correlations. Bonferroni corrections for multiple comparisons were applied to nodal metrics. Due to the importance of M1, a separate analysis focused only on the nodal properties of the precentral gyrus was conducted with a *P* < 0.05 criterion. Statistical analyses were performed in SPSS, version 23.0 (IBM Corporation, Armonk, NY). Two-tailed significance thresholds of *P* < 0.05 were applied.

## Results

### Sample Characteristics

**Figure 2:**
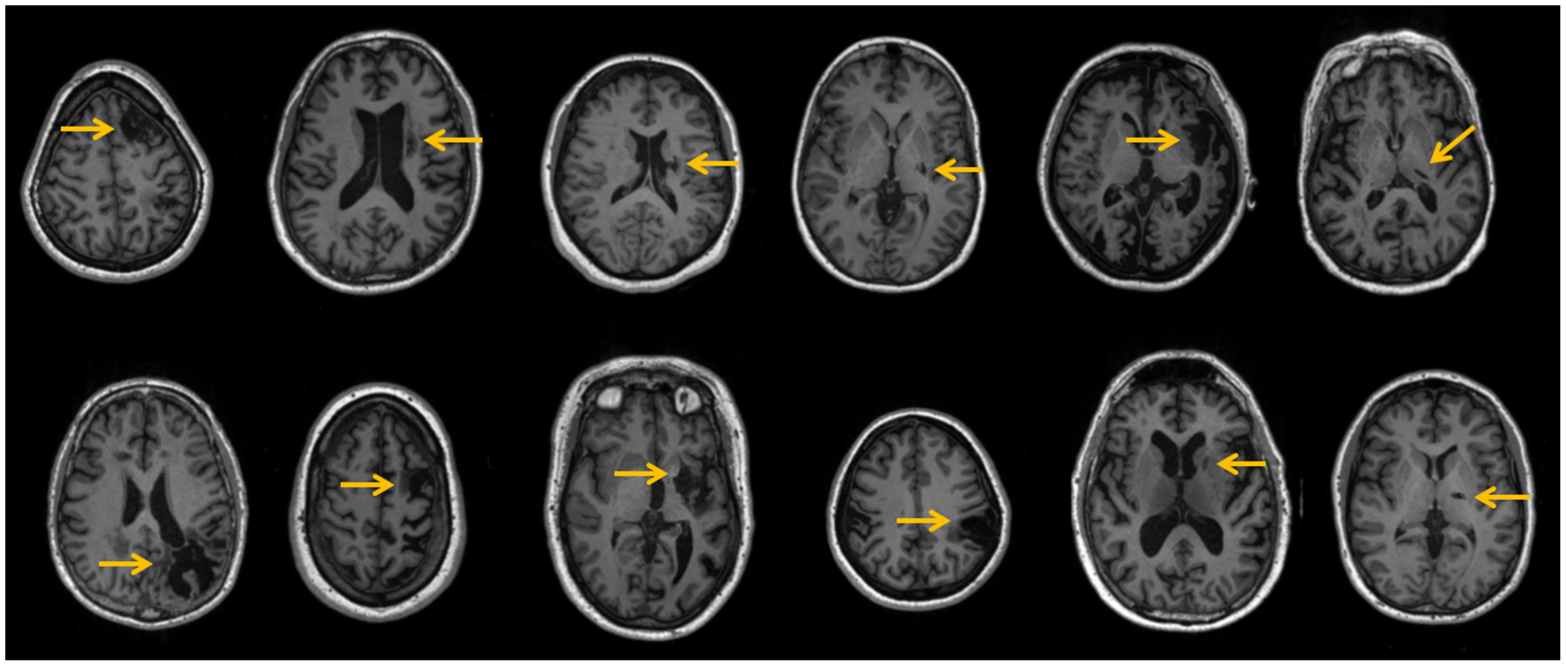
Patients' stroke lesions (yellow arrows) on T1-weighted anatomical slices.

The CSP and HC groups were statistically similar with respect to age and gender representation. The spatial distribution of stroke lesions for all CSPs is depicted in Figure 2. Six patients had subcortical strokes in the putamen, and two had strokes in the insular cortex. The rest of the observed lesions were indicative of neocortical strokes in the frontal, temporal, superior, occipital, and parietal lobes.

All 12 CSPs had clinically significant right-sided motor impairment. They had a mean FM-UE score (±SD) of 27.2 ± 7.4; 2 CSPs were classified as having severe impairment and 10 were classified as having moderate impairment (33). The two patients with severe impairment exhibited spasticity with total ARAT scores of 6 and 3. They were found to have significant variability in both time after stroke (65 ± 52 months) and stroke size (2,422 ± 2,308 mm^3^).

### ROI Analysis

**Figure 3:**
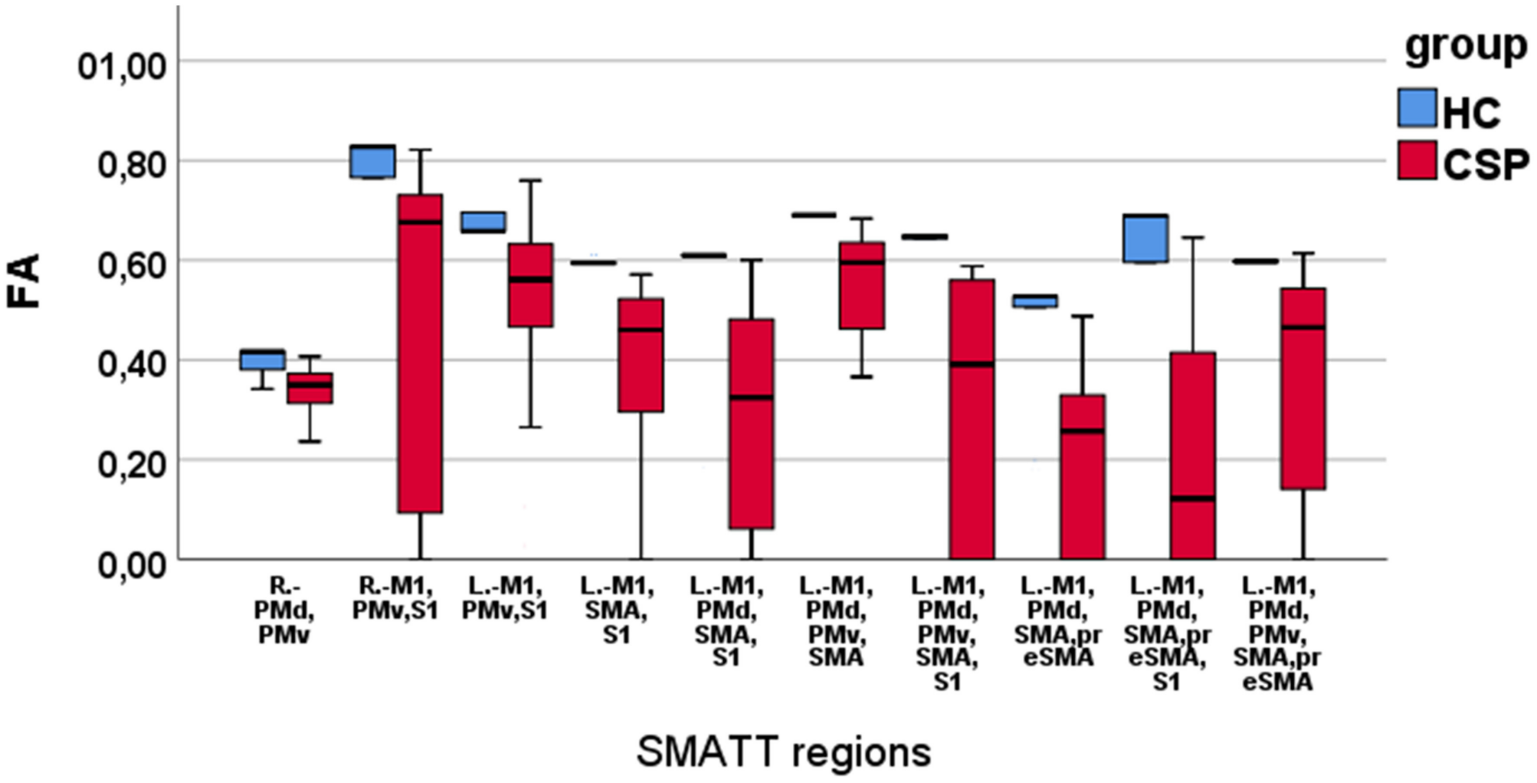
Boxplots of FA values in SMATT regions with significant differences between HC and CSP group. Note that the HCs have higher and less scattered FA values compared with the CSPs.

Mann–Whitney *U*-test indicated that the CSP group had smaller FA values than HCs in the following SMATT regions: L.-M1,PMv,S1; L.-M1,SMA,S1; L.-M1,PMd,SMA,S1; L.-M1,PMd,SMA,preSMA; L.-M1,PMd,PMv,SMA; L.-M1,PMd,PMv,SMA,S1; L.-M1,PMd,SMA,preSMA; L.-M1,PMd,SMA,preSMA,S1; L.-M1,PMd,PMv,SMA,preSMA (Table 1). Decreased FA values for the CSP group were also found in two R. hemisphere regions, namely R.-PMd,PMv and R.-M1,PMv,S1 (Table 1). These results are depicted in Figure 3; note that FA values appear to be more widely dispersed for the CSP group than for the HC group. No MD differences were found between the CSP and HC groups for any SMATT regions. No associations of time after stroke or stroke size with FM-UE scores or SMATT-region diffusion metrics were found.

**Table 1:** FA differences in SMATT regions between HC and CSP groups.

**SMATT region**	**Median FA [minimum, maximum]**		***P***
	**HC group**	**CSP group**	**Mann–Whitney *U-*test**
L.-M1,PMv,S1	0.66 [0.66, 0.76]	0.56 [0.00, 0.76]	5.5 × 10^−4^
L.-M1,SMA,S1	0.59 [0.42, 0.62]	0.46 [0.00, 0.57]	1.1 × 10^−6^
L.-M1,PMd,SMA,S1	0.61 [0.17, 0.64]	0.32 [0.00, 0.60]	1.4 × 10^−6^
L.-M1,PMd,PMv,SMA	0.69 [0.54, 0.74]	0.60 [0.00, 0.68]	1.4 × 10^−6^
L.-M1,PMd,PMv,SMA,S1	0.65 [0.17, 0.65]	0.39 [0.00, 0.59]	8.0 × 10^−7^
L.-M1,PMd,SMA,preSMA	0.52 [0.19, 0.52]	0.25 [0.00, 0.48]	5.8 × 10^−7^
L.-M1,PMd,SMA,preSMA, S1	0.69 [0.19, 0.69]	0.12 [0.00, 0.65]	5.8 × 10^−7^
L.-M1,PMd,PMv,SMA, preSMA	0.60 [0.22, 0.64]	0.47 [0.00, 0.61]	1.4 × 10^−5^
R.-PMd,PMv	0.42 [0.34, 0.42]	0.35 [0.06, 0.40]	1.5 × 10^−6^
R.-M1,PMv,S1	0.83 [0.02 0.83]	0.67 [0.00, 0.82]	1.2 × 10^−6^

*CSP, chronic stroke patient; FA, fractional anisotropy; HC, healthy control; L, left; M1, primary motor cortex; PMd, dorsal premotor cortex; PMv, ventral premotor cortex; R. right; S1, primary somatosensory cortex*.

### LASSO Regression

LASSO regression analysis identified a few FM-UE score predictors among the mean SMATT-region diffusion metrics (Table 2). Regarding FA, bootstraping revealed that among five potential LASSO predictors identified, only the L.-M1,SMA region was significant (adjusted *R*^2^ = 0.773) (Table 2). On the other hand, the mean L.M1,PMv region MD and Left-PMv,SMA region MD values emerged as significant LASSO predictors of FM-UE score (adjusted *R*^2^ = 0.844) (Table 2).

**Table 2:** SMATT regions whose fractional anisotropy or mean diffusivity were LASSO regression predictors of Fugl-Meyer upper extremity scale.

**Diffusion metric**	**SMATT regions (with non-zero LASSO coefficients)**	**LASSO regression results**		
		**Coefficient**	**Standard error**	***P-*value**
Fractional anisotropy	R.-M1,PMv,S1	−0.227	0.149	0.153
	R.-M1,PMv,SMA,S1	−0.059	0.125	0.803
	L.-PMv	0.252	0.147	0.121
	L.-M1,SMA	0.442	0.144	0.004
	L.-SMA,preSMA	−0.217	0.128	0.123
Mean diffusivity (10^−3^ mm^2^/s)	L.-M1,PMv	−0.411	0.129	0.001
	L.-PMv,SMA	−0.395	0.152	0.021

*R, right; M1, primary motor cortex; PMv, ventral premotor cortex; S1, primary somatosensory cortex; SMA, supplementary motor area; L., left; PMd, dorsal premotor cortex*.

### Graph Analysis

**Figure 4:**
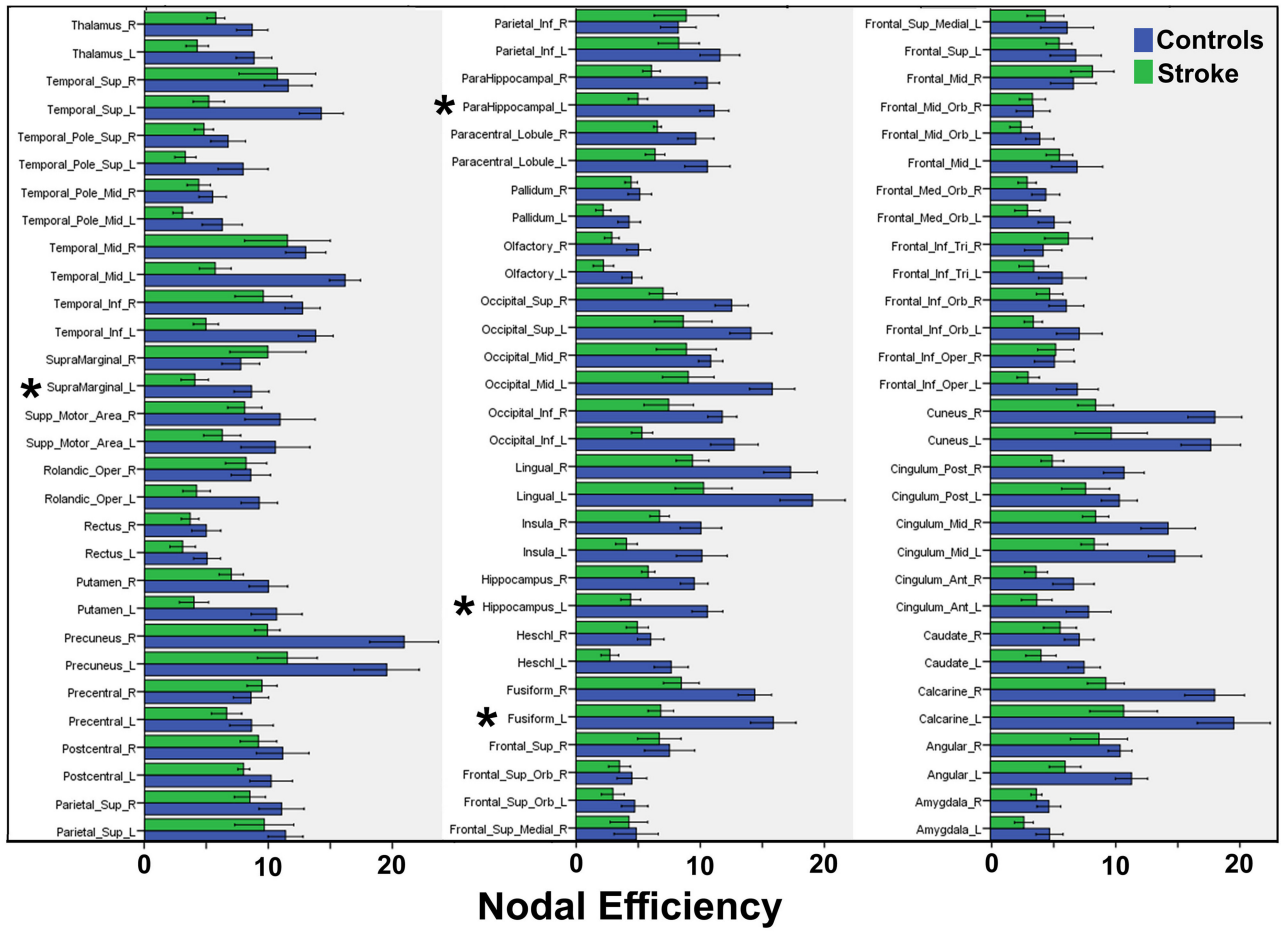
Summary of mean nodal efficiencies. Mean efficiency values are compared between CSP (“Stroke”, green bars) and HC (“Control”, blue bars) groups for 90 AAL regions. Asterisks indicate significant differences, the statistical values for which are reported in Table 3.

Graph analysis revealed that multiple efficiency-related graph metrics differed significantly between the CSP and HC groups (Table 3). Only one global metric, namely network efficiency, differed significantly between the two groups, with CSPs showing a decreased network efficiency relative to HCs. Among the nodal metrics, nodal efficiency of the L. hippocampus, L. parahippocampal gyrus, and L. fusiform gyrus as well as nodal local efficiency of the L. supramarginal gyrus were also found to be decreased in CSPs relative to HCs (Figure 4, Table 3). Betweenness centrality, particularly for the L. precentral gyrus, was found to be increased in the CSP group compared to that of the HC group. No correlations were found between any graph metrics and FM-UE scores.

**Table 3:** Graph metrics that differ significantly between CSPs and HCs.

**Graph metric**	**Mean graph metric value** **±SD**		***P***
**region**	**HC group**	**CSP group**	***T*-test**
Network efficiency	9.7 ± 2.9	6.1 ± 1.7	0.006
**Nodal efficiency**			
L. hippocampus	10.6 ± 4.1	4.4 ± 2.2	0.001
L. parahippocampal gyrus	11.1 ± 4.0	5.0 ± 2.2	0.001
L. fusiform gyrus	15.9 ± 6.3	6.8 ± 2.9	0.001
**Nodal local efficiency**			
L. supramarginal gyrus	38.0 ± 14.1	12.6 ± 11.0	4.6 × 10^−4^
**Betweenness centrality**			
L. precentral gyrus	70.3 ± 68.6	273.5 ± 252.5	0.019

*CSP, chronic stroke patient; HC, healthy control; L., left; SD, standard deviation*.

## Discussion

By combining DTI with graph analysis, we showed that stroke induces topological changes in the structural connectome of the brain. The post-stroke brain was found to be characterized by lower efficiency, or degree of integration, relative to HCs. Notably, CSPs' motor performance scores did not correlate with the aforementioned topological changes but did correlate with DTI-revealed CST abnormalities within specific SMATT-defined segments, and those abnormalities were shown to be independent predictors of motor performance.

Lesion heterogeneity among CSPs has been a major research challenge. In DTI tractography studies specifically, variability in stroke lesion location and size affects major WM tracts in a non-consistent way, thereby precluding detection of a typical outcome for any particular WM tract. Notwithstanding, as was observed in the present sample, post-stroke motor impairment is typically characterized by CST-involved lesions (5), which connect cortical motor regions to neurons in the spinal cord and thus constitute the main descending pathway for movement related information.

In CSPs, CST injury can be the result of direct stroke damage or Wallerian degeneration, defined as anterograde breakdown of myelin with disintegration of its axonal microfilaments (34). The slow evolution of Wallerian degeneration explains why the effects of it become more prominent in the subacute and chronic phases of stroke and why degeneration is often seen in locations distal to the primary stroke lesion (35). Consistent with Wallerian degeneration, our CSPs showed FA differences relative to HCs in CST segments distal to the primary stroke lesions. Noticeably, the significance of the FA difference increased caudally, with the highest values being detected in the lower CST within the brain stem (Table 1). All motor-related tracts converge in the vicinity of the caudal SMATT regions. Thus, regardless of the primary lesion site, Wallerian degeneration affecting these convergence regions is likely to affect motor function. Our finding of CST degeneration being associated with decreased FA is consistent with prior stroke studies that focused exclusively on FA (5, 10, 13).

Our results indicate that the common neuroimaging approach of studying CST pathology by analyzing only the voxels that constitute the middle of the CST tract, known as the skeleton, may be suboptimal, especially in the acute or subacute phases of stroke (10). Although diffusion metrics are very sensitive to pathological changes, they tend to be non-specific and their interpretation is challenging. Here, we focused on the diffusion metrics of FA and MD because they reflect microstructural changes. FA is an index of microstructural WM integrity related to axonal packing density, axonal diameter, myelinization, neurite density, and orientation distribution (36). Meanwhile, MD is an inverse measure of cellularity that is related to water content in the extracellular space (36).

Relatively few studies have included analyses of multiple diffusion indices, especially in CSPs (5). In a recent study focused on the PLIC and the cerebral peduncle ipsilateral to primary stroke lesions, Mastropietro and colleagues (37) found that stroke was associated with increased MD values. Although high signal intensity at the lesion site in MD maps is a well-known radiological finding in chronic stroke, hyperintensities are not known to be associated with distal WM tracts affected by Wallerian degeneration (16). It has been hypothesized that such negative findings for MD values, and the apparent lesser sensitivity of MD to Wallerian degeneration relative to FA, may be consequent to neuroprotective mechanisms mediated by the infiltration of astrocytes, which are relatively resilient to glutamate excitotoxicity during a brain stroke (16, 38, 39). Our regression analysis showing MD associations with FM-UE scores do not support this hypothesis. Conversely, it may be that MD differences are only detectable when a few isolated regions are assessed, and such differences may not survive the application of conservative Bonferroni correction to analyses of 60 SMATT regions.

Decreased FA, relative to HCs, was detected in the R.-PMd,PMv and R.-M1,PMv,S1 regions in our CSP group. Interestingly, both regions contain tracts originating from the contralesional PMv, which has never been the focus of chronic stroke studies. Structural reorganization of the contralesional CST has been demonstrated previously and shown to be associated with motor recovery (10, 13, 40). However, there are conflicting reports regarding the role of the contralesional CST in chronic stroke (14, 41). Findings in the contralesional hemisphere could be consequent to diaschisis, which is the reduction of function, metabolism, and perfusion due to secondary degeneration of transcallosal fibers (42).

The typical approach of employing simple correlation analyses to assess relationships between diffusion metrics and motor performance scores (5) restricts the focus to only a few WM regions or tracts, without consideration of their relative importance to motor skills. Here, an advanced regression model was applied to select independent predictors of motor scale scores from among 60 probabilistic SMATT regions. We thus found that FA of the L.-M1,SMA region was the only independent predictor of FM-UE score. The largest portion of the M1,SMA region belongs to the PLIC (71%) and a smaller portion (14%) belongs to the cerebral peduncle. The PLIC is the most popular region of interest for DTI analysis because it contains the majority of CST fibers. However, the PLIC is larger and relatively non-specific compared to a SMATT region. The L.-M1,SMA includes only 0.9% of the PLIC. SMATT regions with larger PLIC components, such as the L.-M1,S1 (16%) and L.-M1 (6%), did not survive the regression analysis. Thus, the common assertion that the PLIC is important for clinical outcomes after stroke owing mainly to its direct connection to M1 needs revisiting. Among the secondary motor areas connected to the PLIC, the SMA—which is already known to be important for self-initiated movements, planning, motor action sequencing, response inhibition, and bimanual movements—might be highly important to motor performance among CSPs as well.

Our LASSO regression analysis revealed that the L.-M1,PMv and L.-PMv,SMA regions are independent predictors of FM-UE score. Both regions belong mainly to the superior corona radiata and are in close proximity to most of the stroke lesions in our sample. The negative Beta coefficients obtained for our LASSO model (Table 2) indicate that poorer motor outcomes were associated with greater MD values, which reflect substantial fiber destruction. These findings suggest that MD is more sensitive to pronounced structural damage proximal to the lesion than to more subtle distal degeneration. In agreement with the F? results, our MD data suggest that the integrity of CST connections to the SMA and PMv may be important for the preservation of motor skills after stroke.

Our graph analysis showed that brain network and local efficiency were reduced in CSPs relative to HCs. Network efficiency quantifies how well-information is exchanged between all nodes of a network and reflects a brain's capacity to integrate communication between distant regions. Local efficiency quantifies the network's resistance to failure when a node is removed. Findings of reduced network efficiency similar to the present result have been reported in prior electroencephalography and functional MRI studies (43–45), suggesting that stroke can result in reduced functional segregation of the brain. In a study employing DTI and complex network analysis, Crofts et al. (24) calculated an efficiency-like measure called communicability and found that it was reduced in areas surrounding lesions.

Similarly, we observed reduced nodal efficiency in areas near (supramarginal gyrus) as well as far (hippocampus, parahippocampal, and fusiform gyrus) from most of the lesions and the motor circuitry. These results indicate that approaches focusing on a particular tract, like the CST, might lead to underestimation of the extent of alterations in brain network topology. In a recent DTI study applying graph theory, Cheng and colleagues detected reduced brain network efficiency in CSPs as well as greater global clustering and modularity, consistent with increased segregation (27). Contrary to the other few DTI studies in the literature (23, 27), we did not detect changes in network metrics of the contralesional hemisphere. This discrepancy could be related to differences in sample populations, differences in the atlases used for node definition, or, more likely, differences in the tractography approach employed (deterministic vs. probabilistic).

In agreement with our findings, both previous DTI stroke studies (23, 27) did not report associations between brain networks metrics and motor scales. Conversely, in a study in which functional connectivity was analyzed and graph theory was applied, increased centralities were observed for several areas, including the ipsilesional M1, SMA, bilateral thalamus, and anterior inferior cerebellum, and reported to be predictive of post-stroke recovery (43). Only when our analysis was restricted to the L. precentral gyrus, avoiding corrections for multiple comparisons, was a similar tendency toward functional segregation revealed as an increase in betweenness centrality. Considering that betweenness centrality is a measure of the amount of influence that a node has over information flow, this result reflects an increase in the importance of the ipsilesional M1 in the brain connectome after a stroke.

It is likely that the lack of a significant association between time after stroke and FM-UE score reflects the limited recovery experienced by chronic-stroke subjects after the 6-month window of rapid functional recovery immediately after a stroke has occurred, during which pronounced plasticity changes are seen (46). Additionally, the small size and heterogeneity of the sample could explain, at least in part, the non-association between stroke lesion size and FM-UE. Although stroke lesion size has been widely used to assess stroke severity and predict patient outcomes, it is only a moderately powered predictor of motor impairment (47) and quality of life (48). Post-stroke lesion location is a stronger predictor of functional recovery than lesion volume (49–55). As expected, disruption of motor function-involved structures, such as the corona radiata, internal capsule, and insula, have been related to worse functional outcome (49–55). Our atlas-based analysis and our results are consistent with the precept that infarct location is fundamentally linked to neurological deficit profile.

Our subjects were relatively young considering that most strokes occur in people who are 65 years old or older. Age is a factor that limits functional recovery in patients with stroke; better outcomes in younger patients than in older patients (56, 57) likely reflect the greater functional plasticity potential of their brains (58). Although there are no published studies on the age-dependence of brain diffusion metrics in stroke patients to the best of our knowledge, similar studies in healthy subjects have revealed region- or tract-specific increases in anisotropy with decreases in diffusivity (59, 60). Notably, aging-related alterations have been detected in the brain structural connectome and have been linked to cognitive decline (61–63). Given these aforementioned findings, it is not safe to extrapolate our findings to more aged stroke patients.

Our study has several limitations worth noting. Firstly, the relatively small sample size might have limited our ability to detect associations statistically, especially in analyses examining the relationship between the brain connectome and motor outcomes. Although our sample size of 12 is a popular choice among highly cited neuroimaging studies (64), it is not theoretically supported because power analysis in the field of brain connectome research has yet to be explored rigorously. Secondly, heterogeneity of lesion size and location divides our subjects into multiple undersized groups, introduce variability in our measurements and compromise the detection power of our statistical analysis. Thirdly, graph analysis results depend heavily on multiple parameters including atlas choice, sparsity threshold, and graph type (binary vs. weighted). Although we used common practices, they may not be optimal for our study population. Fourthly, our results, obtained in a cohort that was younger than most stroke patients, might not be generalized to the general stroke population. Our results should be considered preliminary and our approach exploratory with the aim of demonstrating the potential for using the SMATT atlas and graph-based analysis in studies of the brain connectome in stroke patients. Larger and age-targeted studies, studies focusing on specific brain regions (e.g., putaminal stroke), and studies using alternative atlases or tract detection methods (i.e., probabilistic tractography) are needed to validate our conclusions.

In conclusion, a WM template specific for the sensorimotor cortex was shown to be useful for refining CST degeneration and its relationship with the motor performance of CSPs. Although stroke induces topological changes in the structural connectome of the brain, including changes that reduce efficiency, these changes may be not associated with motor performance. However, DTI analysis revealed that the integrity of tracts connected to M1, as well as those connected to the SMA and PMv, were independent predictors of motor performance.

## Data Availability Statement

The datasets presented in this article are not readily available because data or metadata (e.g., diffusion maps, connectivity matrices) that were newly acquired for the present study will be made available for computer download upon a request to the authors and subject to a formal data sharing agreement to be approved by the Office of Sponsored Research of Massachusetts General Hospital. Requests to access the datasets should be directed to atzika@hms.harvard.edu.

## Ethics Statement

The studies involving human participants were reviewed and approved by Partners Human Research Committee (protocol no. 2005P000570). The patients/participants provided their written informed consent to participate in this study.

## Author Contributions

AT designed and supervised the study. SE and SL acquired and organized the data. LA analyzed the data and wrote the first draft of the manuscript. AT and SL edited and revised the manuscript. All authors read and approved the submitted version.

## Funding

This research was supported by a grant from the National Institute of Neurological Disorders and Stroke (Grant Number 1R01NS105875-01A1) of the National Institutes of Health to AT.

## Conflict of Interest

The authors declare that the research was conducted in the absence of any commercial or financial relationships that could be construed as a potential conflict of interest.

## Publisher's Note

All claims expressed in this article are solely those of the authors and do not necessarily represent those of their affiliated organizations, or those of the publisher, the editors and the reviewers. Any product that may be evaluated in this article, or claim that may be made by its manufacturer, is not guaranteed or endorsed by the publisher.
